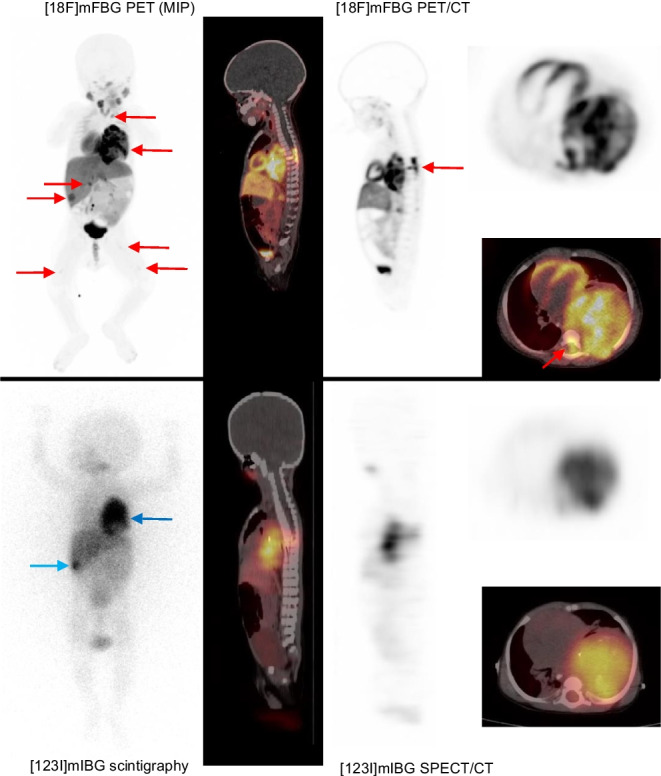# [^18^F]mFBG long axial field of view PET-CT without general anaesthesia reveals concise extension of neuroblastoma in a 9-month-old boy

**DOI:** 10.1007/s00259-023-06160-0

**Published:** 2023-02-28

**Authors:** L. Borgwardt, J. S. Brok, K. F. Andersen, J. Madsen, N. Gillings, M.Ø. Fosbøl, C. L. Denholt, P. S. Wehner, L. H. Enevoldsen, P. Oturai, D. Czyzewska, H. H. Johannesen, L. Højgaard, I. N. Petersen, L. S. Sørensen, C. Schulze, E. S. Saxtoft, F. L. Andersen, B. M. Fischer

**Affiliations:** 1 Department of Clinical Physiology and Nuclear Medicine, University Hospital of Copenhagen, Rigshospitalet, Copenhagen, Denmark; 2 Department of Paediatrics, University Hospital of Copenhagen, Rigshospitalet, Copenhagen, Denmark; 3 Department of Paediatrics, Odense University Hospital, Odense, Denmark

A 9-month-old boy presented with severe acute respiratory compromise. A chest x-ray and CT demonstrated a large thoracic tumour, suspicious of neuroblastoma(NB) with liver metastases, subsequently biopsy-verified. Due to his respiratory status, he was not eligible for general anaesthesia(GA)/sedation. Hence, standard diagnostics with MRI and [^123^I]mIBG-scintigraphy was not feasible [1]. Instead, the tumour extension was assessed with [^18^F]mFBG long axial field of view(LAFOV)-PET/CT (106 cm) without GA/sedation [2, 3], acquired 62 min p.i. of 30 MBq (3 MBq/kg) [4]. CT was performed as ultra-low-dose, followed by PET acquisition of 10 min in list mode, with only 2 min reconstruction required in order to provide a clinically useful image without motion artefacts. The scan showed [^18^F]mFBG uptake in a 9.5 × 7 × 7 cm tumour in the left hemithorax with intraspinal involvement between Th4 and Th8 (red arrows), a left-sided cervical lymph node (red arrow), several liver lesions (red arrows), and in the bone marrow (BM) of Th6 (red arrow) and bilateral femurs (red arrows). Chemotherapy was started immediately. Three days later, the patient was clinically stable, and as part of a prospective study comparing the two scans, [^123^I]mIBG-scintigraphy-SPECT/LDCT could be performed, showing only a large thoracal tumour (blue arrow) and a single certain liver metastasis (blue arrow). The spinal involvement was unclear, and the BM involvement, the cervical lymph node and several liver lesions could not be identified. The BM biopsy taken from the iliac crests was negative using standard staining, but a novel more sensitive GD2-antigen test (oncofoetal glycolipid antigen) verified bilateral BM involvement [5].

In conclusion, a fast [^18^F]mFBG-LAFOV-PET/CT without GA in a critically ill 9-month-old boy revealed a concise extension of neuroblastoma relevant for staging and treatment.